# Persistent short nighttime sleep duration is associated with a greater post-COVID risk in fully mRNA-vaccinated individuals

**DOI:** 10.1038/s41398-023-02334-4

**Published:** 2023-02-01

**Authors:** Pei Xue, Ilona Merikanto, Frances Chung, Charles M. Morin, Colin Espie, Bjørn Bjorvatn, Jonathan Cedernaes, Anne-Marie Landtblom, Thomas Penzel, Luigi De Gennaro, Brigitte Holzinger, Kentaro Matsui, Harald Hrubos-Strøm, Maria Korman, Damien Leger, Sérgio Mota-Rolim, Courtney J. Bolstad, Michael Nadorff, Giuseppe Plazzi, Catia Reis, Rachel Ngan Yin Chan, Yun Kwok Wing, Juliana Yordanova, Adrijana Koscec Bjelajac, Yuichi Inoue, Markku Partinen, Yves Dauvilliers, Christian Benedict

**Affiliations:** 1 Department of Pharmaceutical Biosciences, Molecular Neuropharmacology, Uppsala University, Uppsala, Sweden; 2 Research Programs Unit, Faculty of Medicine, University of Helsinki, Helsinki, Finland; 3 Department of Anesthesiology and Pain Medicine, University Health Network, University of Toronto, Toronto, Ontario Canada; 4 Centre de recherche CERVO/Brain Research Center, École de psychologie, Université Laval, Quebec City, Quebec Canada; 5 Sleep and Circadian Neuroscience Institute, Nuffield Department of Clinical Neurosciences, University of Oxford, Oxford, UK; 6 Department of Global Public Health and Primary Care, University of Bergen, Bergen, Norway; 7 Norwegian Competence Center for Sleep Disorders, Haukeland University Hospital, Bergen, Norway; 8 Department of Medical Sciences, Transplantation and regenerative medicine, Uppsala University, Uppsala, Sweden; 9 Department of Medical Cell Biology, Uppsala University, Uppsala, Sweden; 10 Department of Medicine, Feinberg School of Medicine, Northwestern University, Chicago, IL USA; 11 Department of Medical Sciences, Neurology, Uppsala University, Uppsala, Sweden; 12 Department of Biomedical and Clinical Sciences, Linköping University, Linköping, Sweden; 13 Sleep Medicine Center, Charite University Hospital Berlin, Berlin, Germany; 14 Department of Psychology, Sapienza University of Rome, Roma, Lazio Italy; 15 IRCCS Fondazione Santa Lucia, Roma, Italy; 16 Institute for Consciousness and Dream Research; Medical University of Vienna, Wien, Postgraduate Sleep Coaching, Vienna, Austria; 17 Department of Clinical Laboratory, National Center Hospital, National Center of Neurology and Psychiatry, Kodaira, Japan; 18 Department of Otorhinolaryngology, Akershus University Hospital, Lørenskog, Norway; 19 Institute of Clinical Medicine, University of Oslo, Oslo, Norway; 20 Department of Occupational Therapy, Faculty of Health Sciences, Ariel University, Ariel, Israel; 21 Sleep and Vigilance Center, Hopital Hotel-Dieu de Paris, Paris, France; 22 VIFASOM (EA 7331 Vigilance Fatigue Sommeil et Santé Publique), Universite de Paris, Paris, France; 23 Brain Institute, Onofre Lopes University Hospital, and Physiology and Behavior Department, Federal University of Rio Grande do Norte, Natal, Brazil; 24 Department of Psychology, Mississippi State University, Mississippi State, Mississippi, USA; 25 IRCCS Istituto Delle Scienze Neurologiche di Bologna, Bologna, Italy; 26 Department of Biomedical, Metabolic and Neural Sciences, University of Modena and Reggio Emilia, Modena, Italy; 27 Universidade Católica Portuguesa, Católica Research Centre for Psychological - Family and Social Wellbeing, Lisbon, Portugal; 28 Instituto de Medicina Molecular João Lobo Antunes, Faculdade de Medicina de Lisboa, Universidade de Lisboa, Lisboa, Portugal; 29 Instituto de Saúde Ambiental, Faculdade de Medicina, Universidade de Lisboa, Lisboa, Portugal; 30 Li Chiu Kong Family Sleep Assessment Unit, Department of Psychiatry, Faculty of Medicine, The Chinese University of Hong Kong, Hong Kong SAR, China; 31 Institute of Neurobiology, Bulgarian Academy of Sciences, Sofia, Bulgaria; 32 Institute for Medical Research and Occupational Health, Zagreb, Croatia; 33 Department of Somnology, Tokyo Medical University, Tokyo, Japan; 34 Japan Somnology Center, Institute of Neuropsychiatry, Tokyo, Japan; 35 Department of Clinical Neurosciences, University of Helsinki Clinicum Unit, Helsinki, Finland; 36 Helsinki Sleep Clinic, Terveystalo Healthcare Services, Helsinki, Finland; 37 Sleep-Wake Disorders Center, Department of Neurology, Gui-de-Chauliac Hospital, Institute for Neurosciences of Montpellier INM, INSERM, University of Montpellier, Montpellier, France

**Keywords:** Diseases, Physiology

## Abstract

Short nighttime sleep duration impairs the immune response to virus vaccination, and long nighttime sleep duration is associated with poor health status. Thus, we hypothesized that short (<6 h) and long (>9 h) nighttime sleepers have a higher post-COVID risk than normal nighttime sleepers, despite two doses of mRNA vaccine (which has previously been linked to lower odds of long-lasting COVID-19 symptoms). Post-COVID was defined as experiencing at least one core COVID-19 symptom for at least three months (e.g., shortness of breath). Multivariate logistic regression adjusting for age, sex, BMI, and other factors showed in 9717 respondents (age span 18–99) that two mRNA vaccinations lowered the risk of suffering from post-COVID by about 21% (*p* < 0.001). When restricting the analysis to double-vaccinated respondents (*n* = 5918), short and long sleepers exhibited a greater post-COVID risk than normal sleepers (adjusted OR [95%-CI], 1.56 [1.29, 1.88] and 1.87 [1.32, 2.66], respectively). Among respondents with persistent sleep duration patterns during the pandemic compared to before the pandemic, short but not long sleep duration was significantly associated with the post-COVID risk (adjusted OR [95%-CI], 1.59 [1.24, 2.03] and 1.18 [0.70, 1.97], respectively). No significant association between sleep duration and post-COVID symptoms was observed in those reporting positive SARS-CoV-2 test results (*n* = 538). Our findings suggest that two mRNA vaccinations against SARS-CoV-2 are associated with a lower post-COVID risk. However, this protection may be less pronounced among those sleeping less than 6 h per night. Our findings warrant replication in cohorts with individuals with confirmed SARS-CoV-2 infection.

## Introduction

SARS-CoV-2 vaccines, including mRNA vaccines, have become a powerful strategy for the global control of the COVID-19 pandemic as they can elicit long-persisting antibody responses [1]. In addition to lower odds of infection, hospitalization, and death [2], SARS-CoV-2 vaccines hold some promise in reducing the risk of long-lasting COVID symptoms. For example, a previous study showed that the likelihood of long-lasting COVID symptoms decreased after SARS-CoV-2 vaccination, and evidence suggested sustained improvement after a second dose [3]. As suggested by a recent large-scale survey study involving 76,422 participants, core long-lasting COVID symptoms include chest pain, difficulties with breathing, pain when breathing, painful muscles, ageusia or anosmia, tingling extremities, lump in the throat, feeling hot and cold alternately, heavy arms or legs, and general tiredness [4].

In humans, sleep loss negatively impacts the immune system [5]. Individuals who do not get enough sleep are more susceptible to illness and have a more challenging time fighting off infections [6–9]. In addition, short nighttime sleep duration may compromise the development of adaptive immunity. For example, participants sleep-deprived in the night after vaccination against the hepatitis virus A exhibited lower numbers of antigen-specific T helper cells and reduced antibody titers in the blood [10, 11]. Furthermore, in a study with 25 healthy young men, antibody titers after immunization against the seasonal flu were less than half among those whose bedtime was restricted to four hours for six nights compared to a group of subjects who maintained their usual bedtime [12]. These findings highlight the importance of adequate sleep for maintaining a healthy immune system and coping with virus infections.

Given that short nighttime sleep duration is associated with an impaired immune response to virus vaccination with possible adverse implications for disease recovery [7, 8, 10–15], we hypothesized that the risk of long-lasting COVID symptoms would be higher among fully vaccinated individuals with habitual short nighttime sleep duration (defined as <6 h per night) compared to those with normal (6–9 h per night) nighttime sleep duration [16–18]. Long nighttime sleep duration, often defined as more than nine hours of sleep per night [16–18], is associated with poor health status [19–22]. For example, similar to short sleep duration, long sleep has been associated with increased reports of head and chest colds in a nationally representative sample of US adults [23]. Thus, we hypothesized that two mRNA vaccinations against SARS-CoV-2 would confer less protection against long-lasting COVID symptoms in long nighttime sleepers (defined as >9 h per night) than in normal nighttime sleepers. We analyzed data from several thousands of participants who responded to a standardized internet-based survey to test these hypotheses.

## Materials and methods

### Study design and participants

The present analyses relied on data from the second International Covid Sleep Study survey (ICOSS-2). The second survey aimed to investigate the interplay between sleep-wake patterns, COVID-19 disease severity, and post-infection symptoms in the general population. More information about the first and second waves of ICOSS can be found in [24] and [25].

Data collection for ICOSS-2 took place between May and December 2021. The survey was translated into the following languages: German, Portuguese, English, French, Bulgarian, Croatian, Chinese, Finnish, Hebrew, Italian, Japanese, Norwegian, and Swedish. The most used online platforms were RedCap and Qualtrics. Potential participants were solicited, for example, by informing about the study on the university web pages, newspapers, television, Facebook, or Twitter. All responders were anonymous volunteers aged 18 years or older. In total, 16899 participants responded to ICOSS-2. Following exclusions detailed in Supplemental Table S1, 9717 participants (58%) remained available for testing the hypothesis that two doses of mRNA vaccines lower the risk of suffering from at least one core long-lasting COVID symptom (definition below). Similar to previous studies [16–18], habitual self-reported nighttime sleep duration was divided into three categories: short (<6 h per night), normal (6–9 h per night), and long (>9 h per night) nighttime sleep duration. The hypothesis that the risk of post-COVID varies by nighttime sleep duration was analyzed in 5918 fully-vaccinated participants.

This research was conducted according to the Declaration of Helsinki, all countries obtained ethical approval or exemptions in keeping with national research governance and regulations (Supplemental Table S2). To access the questions, participants had to give their consent to participate, and they needed to be at least 18 years of age. Participants did not receive any monetary compensation.

### mRNA vaccination status

At least two SARS-CoV-2 vaccinations have been associated with sustainable improvements of long-lasting COVID-19 symptoms in a cohort study from Italy [26]. Thus, in the present study, participants were divided into two mRNA vaccination groups: two SARS-CoV-2 vaccinations vs. 0–1 SARS-CoV-2 vaccination at the time of the survey. We restricted our analysis to the mRNA vaccines (Moderna and BioNTech/Pfizer) as most of our participants were inoculated with them (88%). When starting the survey in May 2021, a third SARS-CoV-2 vaccination was uncommon. Thus, we did not survey whether participants had received more than two SARS-CoV-2 mRNA vaccinations.

### Definition of long-lasting COVID symptoms

Long-lasting COVID-19 symptoms were deemed present if a respondent reported at least one of the following symptoms lasting for at least 3 months at the time of the survey (in the following named post-COVID): shortness of breath or difficulty breathing and/or chest pain, joint pain (arthralgia) and/or muscle pain, muscle aches, post-exertional malaise (prolonged weakness or reduced functionality after exertion), problems with sweating and/or trouble tolerating cold/heat, loss of smell and/or taste, and feverishness and/or flu-like symptoms such as sore throat and runny nose.

We focused on these symptoms because they are similar to those described in previous large-scale studies [4]. By examining these symptoms, we were able to identify cases of post-COVID in our study population. Although fatigue represents one of the core long-lasting COVID symptoms [4], we did not consider it in the definition of post-COVID as short and long sleepers may suffer from fatigue irrespective of a previous SARS-CoV-2 infection [27].

### Potential confounders

The following participants’ characteristics were included as confounders in the fully adjusted model: age, gender, ethnicity, current smoking status (with the following response options: never or less than once per month, less than once per week, 1–5 days per week, every day or almost daily), weekly alcohol consumption (divided into three categories for the analysis: no consumption of alcohol, moderate consumption defined as <12 bottles of beer/cider (33cl), <12 glasses of wine (12cl), or <12 shots of strong sprit (4cl) per week, and excessive alcohol consumption defined as ≥12 bottles of beer/cider, ≥12 glasses of wine, or ≥12 shots of strong sprit per week), highest educational level (binary: those with vs. without a degree from the university, college or above), time elapsed since first vaccination (divided into three categories for the analysis: less than 1 month, 1–6 months, and more than 6 months; only included in the adjusted analysis when investigating whether odds of post-COVID vary by habitual nighttime sleep duration), marital status (divided into three categories for the analysis: single, married/in relationship and divorced/separated/ widowed), living area (binary: urban vs. rural), weekly physical activity level score (description of the score can be found in Supplementary Table S3), weight status (binary: BMI ≥30 kg/m^2^ vs. <30 kg/m^2^), arterial hypertension status (binary: yes vs. no), presence of diseases of the circulatory system (binary: at least one vs. none, atrial fibrillation, heart failure, other heart conditions, and stroke), presence of diabetes mellitus (binary: at least one vs. none, type 1 diabetes or type 2 diabetes), presence of diseases of the respiratory system (binary: at least one vs. none, asthma and chronic obstructive pulmonary disease), presence of neuropsychiatric disorders (binary: at least one vs. none, problems of attention or concentration/ attention deficit hyperactivity disorder, motor disorder, depression, cognitive disorders, migraine, anxiety/panic disorder), and presence of other diseases (binary: at least one vs. none, autoimmune disease, immunosuppressive treatment, allergy, chronic pain/fibromyalgia, chronic fatigue, kidney failure, cancer/use of cytostatic medication).

### Statistical analysis

Data are shown as mean ± SD unless otherwise stated. Comparisons of group characteristics were performed by Chi-Square testing for categorical variables and generalized linear models for continuous variables. We performed logistic regression analyses to test our hypotheses (SPSS 26.0, IBM Corp., Armonk, NY, USA). We report unadjusted and adjusted odds ratios and 95%-confidence intervals. In the analysis investigating the odds of post-COVID in respondents who received two doses of mRNA, those with no vaccination or one mRNA vaccination were used as the reference group. When examining whether the odds of post-COVID vary by habitual nighttime sleep duration among fully-vaccinated respondents, the normal nighttime sleep duration group (6–9 h per night) was used as the reference group.

We performed sensitivity analyses to investigate potential bias. First, we re-ran the analysis dividing nighttime sleep duration into less than 7 h, 7–9 h (which is often mentioned as the optimal sleep duration range) [27, 28], and more than 9 h per night. Second, we re-ran the analysis in those who got fully vaccinated and stayed within the same sleep duration category (e.g., they were short nighttime sleepers before the pandemic and at the time of the survey) (*n* = 5168). Finally, we restricted our analysis to those reporting that they had tested positive at the time of the survey (*n* = 538). Overall, a two-sided *p* < 0.05 was regarded as statistically significant.

## Results

Fully vaccinated respondents exhibited lower odds of suffering from post-COVID than those who were unvaccinated and those who underwent just one mRNA vaccination (unadjusted OR [95%-CI], 0.62 [0.56, 0.67]; adjusted OR [95%-CI], 0.79 [0.71, 0.89]; *p* < 0.001). When comparing the odds of experiencing post-COVID symptoms between single-vaccinated and unvaccinated respondents, no significant difference was found (adjusted OR [95%-CI] for single-vaccinated, 1.13 [0.95, 1.34]; *p* = 0.169). Supplementary Table S4 summarizes the characteristics of the vaccination sample.

When comparing characteristics of double-vaccinated short, normal, and long sleepers, we found that long sleepers were younger and more often reported female gender than the other two sleep duration groups. Furthermore, those reporting short sleep duration exhibited the lowest weekly physical activity score and more often reported unhealthy weight. More characteristics for the sleep duration groups can be found in Table 1.

**Table 1:** ICOSS-2 characteristics of respondents who received two doses of mRNA vaccines split by habitual nighttime sleep duration.

	Habitual nighttime sleep duration			Generalized LM	Chi2-test	
Characteristic	<6 h	6–9 h	>9 h	df; Wald Chi2	df; Pearson Chi2	*P* value
Responders, *n* (%)	990 (16.7)	4733 (80.0)	195 (3.3)	--	--	--
Age, mean (SD)	46.4 (16.3)	45.2 (17.6)	42.1 (17.8)	2; 11.251	--	0.004
Female, *n* (%)	615 (62.1)	2964 (62.6)	145 (74.4)	--	2; 11.386	0.003
Ethnicity, *n* (%)				--	4; 70.966	<0.001
*Caucasian/White*	456 (46.1)	2323 (49.1)	148 (75.9)			
*Asian*	433 (43.7)	2061 (43.5)	32 (16.4)			
*other*	101 (10.2)	349 (7.4)	15 (7.7)			
Smoking status, *n* (%)				--	6; 29.326	<0.001
*never or less than once per month*	760 (76.8)	3832 (81.0)	136 (69.7)			
*less than once per week*	25 (2.5)	119 (2.5)	8 (4.1)			
*1–5 days per week*	23 (2.3)	126 (2.7)	4 (2.1)			
*every day or almost daily*	182 (18.4)	656(13.9)	47 (24.1)			
Weekly alcohol consumption, *n* (%)				--	4; 29.079	<0.001
*not at all*	410 (41.4)	1624 (34.3)	56 (28.7)			
*moderate*	540 (54.5)	2975 (62.9)	134 (68.7)			
*excessive*	40 (4.0)	134 (2.8)	5 (2.6)			
Weekly PAL score, mean (SD)	1.6 (2.2)	1.9 (2.3)	2.0 (2.2)	2; 8.831	--	0.012
Marital status, *n* (%)					4; 24.091	<0.001
*single*	291 (29.4)	1456 (30.8)	59 (30.3)			
*married /in relationship*	572 (57.8)	2894 (61.1)	123 (63.1)			
*divorced or separated/widowed*	127 (12.8)	383 (8.1)	13 (6.7)			
Living area, *n* (%)				--	2; 11.150	0.004
*urban*	548 (55.4)	2688 (56.8)	133 (68.2)	--		
BMI ≥ 30 kg/m^2^, n (%)	170 (17.2)	563 (11.9)	30 (15.4)	--	2; 21.412	<0.001
University, College or above, *n* (%)	553 (55.9)	3107 (65.6)	124 (63.6)	--	2; 34.023	<0.001
Test-positive for SARS-CoV-2, *n* (%)	112 (11.3)	405 (8.6)	21 (10.8)	--	2; 8.213	0.016
Presence of						
*diseases of the circulatory system, n (%)*	250 (25.3)	938 (19.8)	53 (27.2)	--	2; 19.281	<0.001
*diabetes mellitus, n (%)*	216 (21.8)	689 (14.6)	35 (17.9)	--	2; 32.950	<0.001
*diseases of respiratory system, n (%)*	120 (12.1)	423 (8.9)	34 (17.4)	--	2; 22.970	<0.001
*neuropsychiatric disorders, n (%)*	425 (42.9)	1579 (33.4)	125 (64.1)	--	2; 101.803	<0.001
*other diseases, n (%)*	457 (46.2)	1887 (39.9)	121 (62.1)	--	2; 47.864	<0.001
*hypertension, n (%)*	198 (20.0)	752 (15.9)	43 (22.1)	--	2; 13.925	<0.001

Group comparisons were performed by Chi-Square testing for categorical variables and generalized linear models for continuous variables.

*df* degree of freedom, *LM* linear model, *PAL* physical activity level.

When running the logistic regression to investigate the association of sleep duration and post-COVID in double-vaccinated individuals, we found that those reporting short and long nighttime sleep had 1.56 to 1.87-fold higher odds of post-COVID than normal nighttime sleepers (Table 2; the strength and direction of the association between confounders and the risk of post-COVID are detailed in Supplemental Table S5). Associations between the risk of experiencing the individual core post-COVID symptoms and nighttime sleep duration are summarized in Table 3.

**Table 2:** Association of habitual nighttime sleep duration with post-COVID among those receiving two mRNA vaccinations.

Nighttime sleep duration	*n*	Post-COVID, n (%)	OR [95%-CI]	
			Unadjusted	Adjusted^a^
6–9 h	4733	930 (19.6)	1 [ref]	1 [ref]
<6 h	990	306 (30.9)	1.83 [1.57, 2.13]	1.56 [1.29, 1.88]
>9 h	195	84 (43.1)	3.10 [2.31, 4.15]	1.87 [1.32, 2.66]

^a^Adjusted for: participants’ age, gender, ethnicity, test positivity status, time elapsed since first mRNA vaccination, smoking status, weekly alcohol consumption, educational level, marital status, living area, the weekly physical activity level status score, presence of obesity/hypertension/diseases of the circulatory system/diabetes mellitus/diseases of respiratory system/neuropsychiatric disorders/other diseases.

**Table 3:** Association of habitual nighttime sleep duration with individual post-COVID symtpoms among those receiving two mRNA vaccinations.

Experienced for at least three months	Finding		6–9 h	<6	>9 h
			*n* = 4733	*n* = 990	*n* = 195
Shortness of breath or difficulty breathing and/or chest pain	Proportion		6.2%	10.9%	19.5%
	OR [95%-CI]	Unadjusted	1 [ref]	1.85 [1.47, 2.34]	3.65 [2.51, 5.30]
		Adjusted^a^		1.39 [1.15, 1.67]	2.26 [1.76, 2.91]
Joint pain (arthralgia) and/or muscle pain, muscle aches	Proportion		11.6%	21.1%	25.1%
	OR [95%-CI]	Unadjusted	1 [ref]	2.04 [1.71, 2.44]	2.56 [1.83, 3.59]
		Adjusted^a^		1.67 [1.44, 1.95]	1.87 [1.48, 2.37]
Post-exertional malaise	Proportion		7.8%	12.5%	28.2%
	OR [95%-CI]	Unadjusted	1 [ref]	1.69 [1.36, 2.10]	4.63 [3.33, 6.43]
		Adjusted^a^		1.52 [1.27, 1.80]	2.60 [2.04, 3.32]
Problems of sweating and /or trouble of tolerating cold/heat	Proportion		6.5%	12.5%	16.9%
	OR [95%-CI]	Unadjusted	1 [ref]	2.07 [1.66, 2.58]	2.93 [1.98, 4.34]
		Adjusted^a^		1.73 [1.45, 2.07]	1.79 [1.38, 2.32]
Loss of smell and/or taste	Proportion		2.7%	3.9%	8.2%
	OR [95%-CI]	Unadjusted	1 [ref]	1.46 [1.02, 2.11]	3.22 [1.88, 5.53]
		Adjusted^a^		0.99 [0.75, 1.29]	2.16 [1.55, 3.02]
Feverishness and/or flu-like symptoms, such as sore throat, runny nose etc.	Proportion		2.6%	4.2%	9.2%
	OR [95%-CI]	Unadjusted	1 [ref]	1.63 [1.14, 2.33]	3.74 [2.23, 6.28]
		Adjusted^a^		1.37 [1.08, 1.75]	2.65 [1.98, 3.54]

^a^Adjusted for: participants’ age, gender, ethnicity, test positivity status, time elapsed since first mRNA vaccination, smoking status, weekly alcohol consumption, educational level, marital status, living area, the weekly physical activity level score, presence of obesity/hypertension/diseases of the circulatory system/diabetes mellitus/diseases of respiratory system/neuropsychiatric disorders/other diseases.

### Sensitivity analyses

Our main findings were confirmed when dividing nighttime sleep duration into less than 7 h, 7–9 h, and more than 9 h per night (Supplementary Table S6). When restricting the analysis to those with persistent sleep duration patterns, we found that short nighttime sleep duration remained significantly associated with the post-COVID risk (adjusted OR [95%-CI], 1.59 [1.24, 2.03]; *p* < 0.001). In contrast, long nighttime sleep duration did not reach significance in the fully adjusted model (adjusted OR [95%-CI], 1.18 [0.70, 1.97]; *p* = 0.539) (Table 4). At the time of the survey, 538 respondents indicated that they had tested positive for SARS-CoV-2. As shown in Table 5, the proportion of post-COVID cases was higher among short and long sleepers compared to normal sleepers. However, our analysis did not find a significant association between sleep duration and the odds of experiencing post-COVID. Specifically, neither the unadjusted nor adjusted logistic regression revealed significant differences in the odds of suffering from post-COVID among the different sleep duration categories.

**Table 4:** Association of habitual nighttime sleep duration with post-COVID among those who received two mRNA vaccine doses and exhibited persistent sleep duration patterns between before the pandemic and during the pandemic.

Nighttime sleep duration	*n*	Post-COVID, *n* (%)	OR [95%-CI]	
			Unadjusted	Adjusted^a^
6–9 h	4512	868 (19.2)	1 [ref]	1 [ref]
<6 h	564	143 (25.4)	1.43 [1.16, 1.75]	1.59 [1.24, 2.03]
>9 h	92	30 (32.6)	2.03 [1.31, 3.16]	1.18 [0.70, 1.97]

^a^Adjusted for: participants’ age, gender, ethnicity, test positivity status, time elapsed since first mRNA vaccination, smoking status, weekly alcohol consumption, educational level, marital status, living area, the weekly physical activity level score, presence of obesity/hypertension/diseases of the circulatory system/diabetes mellitus/diseases of respiratory system/neuropsychiatric disorders/other diseases.

**Table 5:** Association of habitual nighttime sleep duration with post-COVID among survey respondents who received two mRNA vaccine doses and reported a positive SARS-CoV-2 test result.

Nighttime sleep duration	*n*	Post-COVID, *n* (%)	OR [95%-CI]	
			Unadjusted	Adjusted^a^
6–9 h	405	192 (47.4)	1 [ref]	1 [ref]
<6 h	112	59 (52.7)	1.24 [0.81, 1.88]	1.03 [0.58, 1.82]
>9 h	21	14 (66.7)	2.22 [0.88, 5.61]	1.96 [0.59, 6.55]

^a^Adjusted for: participants’ age, gender, ethnicity, time elapsed since first mRNA vaccination, smoking status, weekly alcohol consumption, educational level, marital status, living area, the weekly physical activity level score, presence of obesity/hypertension/diseases of the circulatory system/diabetes mellitus/diseases of respiratory system/neuropsychiatric disorders/other diseases.

Similar to the results seen in the fully-vaccinated population, we observed that habitual short and long nighttime sleep duration was associated with a greater post-COVID risk among unvaccinated and those who had undergone one mRNA vaccination at the time of the survey (*n* = 3734; adjusted OR [95%-CI], short nighttime sleep duration: 1.91 [1.50, 2.42]; long nighttime sleep duration: 2.07 [1.54, 2.78]; *p* < 0.001).

## Discussion

Using data from 9717 respondents who partook in an international survey in 2021, we show that two vaccinations with mRNA vaccines against SARS-CoV-2 were associated with 21% lower risk of suffering from post-COVID. When restricting our analysis to 5918 subjects who were vaccinated twice at the time of the survey, both short (<6 h per night) and long (>9 h per night) nighttime sleepers exhibited a more significant post-COVID risk than normal nighttime sleepers. However, the association between post-COVID and long nighttime sleep duration was no longer significant when considering people who slept more than 9 h per night before the pandemic and at the time of the survey.

We observed that habitual short nighttime sleepers had 1.56-fold higher odds of post-COVID than normal nighttime sleepers. Our finding that post-COVID was more prevalent among short nighttime sleepers adds to the growing literature suggesting that a lack of sleep impairs immune function and disease recovery. For example, in a study with 153 healthy adults, those sleeping on average less than 7 h over 14 consecutive nights were about three times more likely to develop a cold upon administration of nasal drops containing a rhinovirus than those with ≥8 h [29]. Impaired innate immunity [30], lower production of antigen-specific antibodies [7, 8, 10–15], and lower counts of antigen-specific memory immune cells [10] observed acutely and weeks after experimental sleep loss may explain why short nighttime sleepers in the present study had higher odds of suffering from post-COVID than those sleeping regularly between 6 to 9 h per night, despite being vaccinated with two doses of mRNA vaccine. Of note, reverse causation cannot be ruled out, i.e., those suffering from post-COVID may be more susceptible to short nighttime sleep duration. However, when limiting the analysis to respondents whose sleep duration pattern did not change amid the pandemic compared to before the pandemic, we found that persistent short nighttime sleep duration remained significantly associated with post-COVID risk.

In the main analysis, habitual long nighttime sleep duration was associated with 1.87-fold higher odds of post-COVID than normal nighttime sleep. To our knowledge, no experimental evidence suggests that sleeping longer than recommended (i.e., >9 h per night) may impair immunity. However, habitual long nighttime sleep duration may be a surrogate marker for poor health, rather than a causative factor [31]. Specifically, long nighttime sleep duration has been tied to chronic low-grade inflammation, obesity, type 2 diabetes mellitus, depression, and hypertension [19–22]. Some of these diseases increase the risk for severe COVID-19 and post-COVID. For example, a study with 912 individuals found a persistent and marked association between hypertension and risk for severe COVID-19, even among fully vaccinated patients [32]. Noteworthy, when restricting the analysis to those with persistent sleep duration patterns (i.e., their sleep duration patterns did not change between before the pandemic and during the pandemic), long nighttime sleep duration was no significant predictor of post-COVID in the fully-adjusted model. The latter suggests that habitual long nighttime sleep duration may be a symptom of rather than causing post-COVID.

There are considerations to take into account in the interpretation of our results. First, residual confounding cannot be excluded despite the extensive adjustments made. Second, the data is self-reported, hence susceptible to reporting bias. Third, due to the cross-sectional design, we can neither conclude causality nor determine whether the symptoms experienced by the participants were present before immunization or infection with SARS-CoV-2. Fourth, our primary analysis was performed while adjusting but not stratifying for self-reported SARS-CoV-2 test positivity. Notably, the number of COVID-19 infections might have been higher due to various reasons, such as lack of available SARS-CoV-2 tests, wrong timing of SARS-CoV-2 testing (e.g., antigen test sensitivity peaks four days after illness onset) [33], respondents’ unwillingness to test for SARS-CoV-2, and respondents’ unawareness of being infected with SARS-CoV-2. Previous observations indicate that the number of undetected SARS-CoV-2 cases can be considerable [34]. Still, it is possible that persistent physical symptoms reported by survey respondents might have been associated with the belief in having been infected with SARS-CoV-2, which may explain why 22.3% of the cohort reported post-COVID symptoms [34]. It should also be kept in mind that the efficacy of mRNA vaccinations to lower the odds of post-COVID may be different for newly emerging variants of SARS-CoV-2. Another limitation is that our analysis was restricted to mRNA vaccines, as most respondents were inoculated with them. Thus, it remains unclear how non-mRNA-based SARS-CoV-2 vaccines affect the risk of post-COVID and whether their efficacy varies by habitual sleep duration. Lastly, our findings warrant replication in cohorts of individuals whose SARS-CoV-2 infection has been confirmed by, e.g., PCR testing.

## Conclusions

Two doses of mRNA vaccines against SARS-CoV-2 are associated with a lower post-COVID risk. However, this protection may be less pronounced among those sleeping less than 6 h per night. Hence, increasing the public awareness of sleeping at least 6 h per night may represent a simple measure to reduce the post-COVID risk in fully mRNA-vaccinated individuals. Our findings warrant replication in cohorts with individuals with confirmed SARS-CoV-2 infection.

## Supplementary information

Supplemental material
