# Supplementary material for: Persistent short nighttime sleep duration is associated with a greater post-COVID risk in fully mRNA-vaccinated individuals
Source: Transl Psychiatry. 2023 Feb 1;13:32. doi: 10.1038/s41398-023-02334-4 (PMC9890416; doi:10.1038/s41398-023-02334-4)
Supplement: Supplementary file 1 — Supplemental material [file 41398_2023_2334_MOESM1_ESM.pdf]

Table S1: Exclusions

**Total number of survey responses to ICOSS-2**	**16 899**
Did not answer to the COVID-19 symptom list defined by core symptoms	2837
No report of sleep duration	511
Self-reported sleep duration <3h or >15h	60
No report on age	7
No report on height	1824
No report on weight	25
No report on ethnicity	85
No report on marital status	13
No report on educational status	316
No report on urban-rural classification	4
No report on smoking status	156
No report on alcohol consumption	4
No report on light, moderate, or high physical activity	315
No report on SARS-CoV-2 vaccination information	3
No report on disease history	32
No report on male or female gender	3
Other vaccines were administered than mRNA or participant was unclear about vaccine types	987
**Sample without any vaccine or with mRNA vaccine**	**9717**
Less than two shots	3734
No report on time elapsed since first vaccination	65
**Samples with 2 shots mRNA vaccine**	**5918**

Table S2: Ethical approval data.

Country	*Ethical diary number*	*Notes*
Austria	NA	Due to the anonymous nature of survey collection, the Ethical Board of the Medical University of Vienna, Austria did not require an ethical evaluation or approval.
Brazil	NA	Due to the anonymous nature of survey collection, the Regional Ethical Board did not require an ethical evaluation or approval.
Bulgaria	Protocol Number 46/05.08.2021	Ethics Commission of the Institute of Neurobiology, Bulgarian Academy of Sciences
Canada	2020-151-A-1-R-1 21-05-2021<br>REB#20-5540	The “Comité d’éthique de l’Université Laval » reviewed and approved this research protocol.<br>University Health Network Research Ethics Board, Toronto, Ontario, Canada
China (Hong Kong)	2020.277	Joint Chinese University of Hong Kong-New Territories East Cluster Clinical Research Ethics Committee
Croatia	100-21/21-4 (07.05.2021)	Ethics Committee of the Institute for Medical Research and Occupational Health
Finland	NA	Due to the anonymous nature of survey collection, the Regional Ethical Board did not require an ethical evaluation or approval.
France	NA	Declaration has been made to the CNIL (Comité National Informatique et liberté).<br>Due to the anonymous nature of survey collection, the Ethical Board did not require an ethical evaluation or approval.
Germany	EA1/162/20	Ethics Committee of Charite University Hospital Berlin
Israel	AU-HEA-MK-20210603	The study was approved by the Ariel University Human Research Ethics Committee of the Faculty of Health Sciences
Italy	protocol number: 0000861, April 24, 2021	Institutional Ethics Committee of the Department of Psychology of the Sapienza University of Rome
Japan	No. 198/2020	The ethics committee of the Neuropsychiatric Research Institute, Tokyo, Japan
Norway	NA	Due to the anonymous nature of survey collection, the Regional Ethical Board did not require an ethical evaluation or approval.
Portugal	CES-UCP nº142, May 27th, 2022	The project was approved unanimously by the Life Sciences Ethical Commission of the Portuguese Catholic University.
Sweden	NA	Due to the anonymous nature of survey collection, the Regional Ethical Board did not require an ethical evaluation or approval.
USA	IRB-20-257	Deemed exempt by the Mississippi State University Institutional Review Board.

Notes: Informed consent was obtained where appropriate, and the procedures followed all principles stated by the Declaration of Helsinki.
Abbreviations: NA = non-applicable.

Table S3: Calculation of the weekly physical activity level status

Self-reported physical activity level (h/w)			Ranking	Score for the analysis
Light	*Moderate*	*Vigorous*		
<2-3	<2-3	<2-3	1	0
≥2-3	<2-3	<2-3	2	1
<2-3	≥2-3	<2-3	3	2
<2-3	<2-3	≥2-3	4	3
≥2-3	≥2-3	<2-3	5	4
≥2-3	<2-3	≥2-3	6	5
<2-3	≥2-3	≥2-3	7	6
≥2-3	≥2-3	≥2-3	8	7

Table S4: ICOSS-2 characteristics, split by vaccination status

	Number of SARS-CoV-2 vaccinations		Generalized LM	Chi^2-test	
**Characteristic**	**Fewer than two**	**Two**	**df; Wald Chi^2**	**df; Pearson Chi^2**	**P-value**
Responders, n (%)	3734 (38.4)	5983 (61.6)	--	--	--
Age, mean (SD)	37.9 (14.7)	45.3 (17.4)	1; 461.693	--	<0.001
Female, n (%)	2760 (73.9)	3768 (63.0)	--	2; 124.733	<0.001
Ethnicity, n (%)			--	2; 978.433	<0.001
Caucasian/White	2918 (78.1)	2950 (49.3)			
Asian	482 (12.9)	2565 (42.9)			
other	334 (8.9)	468 (7.8)			
Smoking status, n (%)			--	3; 38.812	<0.001
never or less than once per month	2830 (75.8)	4788 (80.0)			
less than once per week	129 (3.5)	153 (2.6)			
1 - 5 days per week	165 (4.4)	154 (2.6)			
every day or almost daily	610 (16.3)	888 (14.8)			
Weekly alcohol consumption, n (%)			--	2; 16.769	<0.001
not at all	1167 (31.3)	2108 (35.2)			
moderate	2457 (65.8)	3695 (61.8)			
excessive	110 (2.9)	180 (3.0)			
Weekly PAL score, mean (SD)	2.1 (2.4)	1.8 (2.3)	1; 34.663	--	<0.001
Marital status, n (%)			--	2; 74.367	<0.001
single	1460 (39.1)	1831 (30.6)			
married /in relationship	1995 (53.4)	3624 (60.6)			
divorced or separated/widowed	279 (7.5)	528 (8.8)			
Living area, n (%)			--	1; 294.537	<0.001-
urban	2783 (74.5)	3431 (57.3)			
BMI $\geq 30kg/m^2$, n (%)	639 (17.1)	769 (12.9)	--	1; 33.673	<0.001
Highest education, n (%)			--	1; 0.940	0.332
university, college or above	2354 (63.0)	3830 (64.0)			
Habitual nighttime sleep duration, n (%)			--	2; 95.001	<0.001
less than 6 hours per night	461 (12.3)	1003 (16.8)			
6-9 hours per night	3014 (80.7)	4783 (79.9)			
more than 9 hours per night	259 (6.9)	197 (3.3)			
Test-positive for SARS-CoV-2, n (%)	858 (23.0)	538 (9.0)	--	1; 365.549	<0.001
Presence of					
diseases of the circulatory system, n (%)	605 (16.2)	1257 (21.0)	--	1; 34.297	<0.001

diabetes mellitus, n (%)	585 (15.7)	952 (15.9)	--	1; 0.104	0.748
diseases of respiratory system, n (%)	424 (11.4)	580 (9.7)	--	1; 6.846	0.009
neuropsychiatric disorders, n (%)	1831 (49.0)	2155 (36.0)	--	1; 161.024	<0.001
other diseases, n (%)	1893 (50.7)	2487 (41.6)	--	1; 77.385	<0.001
hypertension, n (%)	432 (11.6)	1007 (16.8)	--	1; 50.453	<0.001
Post-COVID	1188 (31.8)	1334 (22.3)	--	1; 108.406	<0.001
Experienced for at least three months					
shortness of breath or difficulty breathing and/or chest pain, n (%)[A]	465 (12.5)	443 (7.4)	--	1; 69.118	<0.001
joint pain (arthralgia) and/or muscle pain, muscle aches, n (%)[B]	646 (17.3)	817 (13.7)	--	1; 24.051	<0.001
post-exertional malaise, n (%)[C]	576 (15.4)	549 (9.2)	--	1; 87.552	<0.001
problems of sweating and /or trouble of tolerating cold/heat, n (%)[D]	434 (11.6)	466 (7.8)	--	1; 40.324	<0.001
loss of smell and/or taste, n (%)[E]	269 (7.2)	185 (3.1)	--	1; 87.188	<0.001
feverishness and/or flu-like symptoms, n (%)[F]	277 (7.4)	185 (3.1)	--	1; 95.130	<0.001

Group comparisons were performed by Chi-Square testing for categorical variables and generalized linear models for continuous variables. Missing data: [A] n=12; Missing data: [B] n=3; Missing data: [C] n=11; Missing data: [D] n=10; Missing data: [E] n=21; Missing data: [F] n=13. *Abbreviations*: df, degree of freedom; LM, linear model; PAL, physical activity level.

Table S5: OR [95%-CI] for the association of confounders with post-COVID among those who received two doses of mRNA vaccines

Confounder	Adjusted OR [95%-CI]
Age (continuous)	1.03 [1.03, 1.04]
Gender identity	
Women	1.69 [1.42, 2.02]
Ethnicity	
Caucasian/White	1
Asian	0.37 [0.30, 0.45]
other	0.92 [0.71, 1.20]
Smoking status	
never or less than once per month	1
less than once per week	1.11 [0.70, 1.78]
1 - 5 days per week	0.96 [0.61, 1.52]
every day or almost daily	1.18 [0.96, 1.45]
Weekly alcohol consumption	
Not at all	1
Moderate	0.96 [0.82, 1.13]
Excessive	1.08 [0.69, 1.71]
Weekly physical activity level status	0.98 [0.94, 1.01]
Marital status	
Married /in relationship	1
Single	0.87 [0.72, 1.05]
Divorced or separated/widowed	0.90 [0.70, 1.15]
Time elapsed since first mRNA vaccination	
1-6 months	1
< 1 month	1.17 [0.77, 1.76]
>6 month	1.02 [0.85, 1.23]
Living area	
Urban	1
Rural	0.88 [0.75, 1.04]
BMI $\geq 30kg/m^2$	1.10 [0.87, 1.40]
Highest education	
University, College or above	1
Primary/elementary/lower secondary school and secondary school/high school/vocational school	1.16 [0.99, 1.36]
Test-positive for SARS-CoV-2	3.32 [2.65, 4.17]
Presence of diseases of the circulatory system	1.75 [1.26, 2.43]
Presence of diabetes mellitus	1.68 [1.35, 2.10]
Presence of diseases of respiratory system	1.53 [1.23, 1.89]
Presence of neuropsychiatric disorders	2.69 [2.29, 3.16]
Presence of other diseases	2.44 [2.08, 2.86]
Presence of hypertension	0.92 [0.65, 1.31]

Supplemental Table S6: Association of habitual nighttime sleep duration with post-COVID among those who received two mRNA vaccinations (defining 7-9 hours of nighttime sleep as the normal sleep duration category)

Nighttime sleep duration	n	Post-COVID, n (%)	OR [95%-CI]	
			Unadjusted Model	Adjusted Model[1]
7-9 hours	3021	575 (19.0)	1 [ref]	1 [ref]
<7 hours	2702	661 (24.5)	1.38 [1.21, 1.56]	1.29 [1.11, 1.50]
>9 hours	195	84 (43.1)	3.22 [2.39, 4.34]	1.95 [1.37, 2.78]

[1]adjusted for: participants' age, gender, ethnicity, test positivity status, time elapsed since first mRNA vaccination, smoking status, weekly alcohol consumption, educational level, marital status, living area, the weekly physical activity level status, presence of obesity/hypertension/diseases of the circulatory system/diabetes mellitus/diseases of respiratory system/neuropsychiatric disorders/other disease
